# Who Reports Low Interactive Psychology Status? An Investigation Based on Chinese Coal Miners

**DOI:** 10.3390/ijerph17103446

**Published:** 2020-05-15

**Authors:** Shuai Han, Hong Chen, Jill Harris, Ruyin Long

**Affiliations:** 1 College of Economic and Management, Shandong University of Science and Technology, Qingdao 266597, China; 2 School of Management, China University of Mining and Technology, Xuzhou 221116, China; longruyinn@163.com; 3 Sustainable Minerals Institute, The University of Queensland, Brisbane 4072, Australia; jill.harris@uq.edu.au

**Keywords:** psychology, interaction, environment, multi-roles, safety, coal mine

## Abstract

In mine safety and health research, psychological issues have always been neglected. This paper aims to identify the psychological perceptions of workers with respect to the mine environment and interpersonal environment across the whole production system. A survey was designed that measured the miners’ demographic details and perceptions of two affect-based interactions; three resource-based interactions for the manager, supervisor, co-worker; and three actual environment interactions. A total of 642 frontline coal miners from six mines located in six provinces in China completed the survey. The main results indicated that that miners reported low psychology status, especially those over 51 years old, with a monthly income of 2000–4000 and junior school education. Second, there was a high proportion of inferior value in environmental interactions. Meanwhile, the miners’ interactions with their co-workers were perceived as the most positive and those with their managers as the least in interpersonal interactions. Third, there were significant differences in sub-dimension interactions (actual environment, resource-based, affect-based interactions) that certainly existed in these interactive roles. Additionally, the dissociated type of miners with manager and supervisor (low resource and affect-based interaction) reached 23.99~24.45%. This study revealed the inner psychological risk factors for safety and health work in coal mines and provides an essential guideline for mining industries.

## 1. Introduction

Safety and health in the mining industry is a global concern. In China, coal accounts for about 60 per cent of total energy production and consumption. Therefore, considering the significant role of coal in the energy structure, safety, and health have been key considerations of the national strategy in recent years [1]. In general, research on mine safety and health have mainly focused on workers’ safety behaviors or the inability to act safely (e.g., behavioral ability, safety knowledge, consciousness, habits, attitudes, and commitments [2,3,4]) and physical health (e.g., physical function, body pain, and chronic disease) [5,6,7]. However, the psychological health of miners is still ignored. In fact, psychological perception is at the root of individual behavior rather than the superficial causes of the loss of knowledge, ability, and so on [8,9].

Insights from ecological psychology are quite valuable for understanding human psychology and behavior in complex systems. It emphasizes that natural and organizational interaction environments are important drivers for psychological perception and further behavior formation [10]. Undeniably, a supportive or positive interaction environment for workers can improve their workplace safety and health performance [11,12]. However, the current contextual environment factors are known to strongly influence the psychological health of miners.

There are several social, geographical, and organizational factors that may hinder their efforts to improve the safety and health situation in Chinese coal mines. One of the factors contributing to the psychological health of miners is the current reorganization and closure of Chinese coal mines. On the other hand, the national policy of curtailing coal production has resulted in a slump in domestic coal production [1,13], which can lead to the unemployment of the miners. Since 2013, approximately 2.2 million mining employees have become unemployed, and 4.44 million people have been reported to be at risk of unemployment [1]. Furthermore, the risks of deeper seam mining due to the reduction of shallow coal seams also lead to the psychological unease of miners [13,14]. In mining enterprises, extremely forced and passive management approaches are still common, which are characterized by high penalties and strict hierarchies and may take the form of bullying, with miners subjected to derision from managers and supervisors. These reasons, collectively, indicate that the miners’ psychological health has been affected, which poses a threat to the safety management of coal mines. To sum up, there is a significant and unfortunate delay in translating the findings from psychological research into the field of safety and health work, especially in the Chinese mining industries [15]. Therefore, practitioners and academics should attach great importance to the psychological dimension [2,14].

Since the above described psychological issues have not been highly valued and explored in mine safety and health research in China, these insights put forward two main research questions: (1) What are the basic environmental components of the psychological interaction of frontline miners? and (2) What are the status and differentiated features of miners’ psychological perception with those environments? Therefore, in order to adequately address these deep-rooted causes of the psychological issues, this study was conducted. The main objective was to understand the perception of the miners’ psychological interaction with the surrounding environment and to reveal why unwanted events and behaviors occur from the psychological point of view.

The rest of the paper is structured as follows. Section 2 is devoted to the literature review. Section 3 describes the research design and survey of the miners’ psychological perceptions. Section 4 presents the analysis and results of the survey. Section 5 provides a discussion of results, and Section 6 presents the conclusions, practical implications, limitations, and suggestions for future research.

## 2. Literature Review

The psychology behind behavior performance considers the workers’ interaction with the surrounding environment in a whole work system (i.e., social, or organizational environment, etc.), rather than the individual or differentiated elements in the system [10,14]. While many categories of interactive psychology environments have been identified in the literature, they can generally be classified into two types: (1) the realistic environment as an objective environment and (2) the interpersonal environment as a subjective environment including the complicated relationship between multi-roles in the work system [10,16,17,18]. As a perspective, the interactive psychological studies of coal mine production can be clearly acknowledged and analyzed based on the frame of the realistic and interpersonal environment.

### 2.1. The Interaction of Workers in the Mine Environment

This study explored the ways that Chinese coal mine workers psychologically perceive related factors in the natural, derivate, and human environments of the mining production system in which they work. Chen [3] and Han et al. [14] argued that the coal mining production system is made up of these three environments. The natural environment refers to those geological characteristics and conditions of the coal mine not influenced by people such as geological conditions, the quality of the coal resources, and the location of mines [19,20]. The derivative environment refers to the external and internal conditions formed by human involvement. External conditions are reflected in broader social processes and are termed as the macro-derivate environment, and examples include supervision from state and government, the social recognition of employment, and the work–family environment [3,21]. In contrast, internal conditions (or the micro-derivate environment) are reflected in the internal organizational design process of the company such as the production, development, management, and cultural systems.

The human environment is at the center of the whole production system. It involves interactions between different worker groups in the organizational structure based on variant positions and responsibilities. There are three main organizational groups: the executive group (i.e., frontline miners); the decision group (i.e., managers); and the supervision group (i.e., supervisors). The frontline miners are involved in coal production. They execute and complete the production task through cooperation with peers. They work across different departments of coal mining such as driving, transport, machine electricity, ventilation, and waterproofing. The miners are the direct implementer of the safe output and are the main sources of unsafe behaviors. The miners occupy a primary place in the organization, whether in total workforce numbers or job function, and their psychological status is the focus of this study. Managers own the decision-making and leadership function of the production and operation across the whole coal mine safety system, guiding and assigning the work of frontline miners. The supervisors have the duties of safety supervision and hazard elimination, mainly charged by the safety supervision department [22]. The supervisors act as important gatekeepers of safe production and are responsible for investigating and processing the hazards in the environment, equipment, and violations of frontline miners as well as the undertaking of the safety education and training of miners. Therefore, the managers and supervisors as well as co-workers have a direct work connection with frontline miners [2,23], which should be taken as interpersonal objects.

### 2.2. The Interpersonal Environment for Psychological Interaction

The quality of the workers’ interactions can be measured according to the way they satisfy innate work-related needs. The individuals’ needs are commonly classified into different categories according to classic theories such as those of Maslow [24], McClelland [25], Alderfer [26], and Herzberg [27]. Clayton Alderfer’s ERG (Existence-Relatedness-Growth) theory condensed Maslow’s five need categories into three, ranging from basic material needs, interpersonal relationship needs, and personal development needs, respectively. As research on human needs and motivations grew, they began to be applied to the field of work and organizational psychology. For example, social exchange theory [28], psychological contract theory [29,30], conservation of resource theory [31], and LMX (Leader-member exchange) theory [32,33]. For this study, we categorized workers’ needs according to whether they were externally or internally oriented. External needs are termed resource-based needs and are characterized by the tangible, transactional, instrumental, and corporeal interaction processes. In contrast, internal needs are termed as affect-based needs and are characterized by intangible, relational, and sensorial interaction processes.

Resource-based needs are further categorized into the task, relatedness, and growth resource needs, informed by existence, relatedness, and growth need from Alderfer’s ERG theory. This study replaces existence needs with task needs because of the characteristic of tasks in a whole production system. The operation of the whole coal production system is task-oriented [3], and workers acquire existence resources (e.g., salary, rewards, other basic materials) depending on the achievement of the task. Hence, the task resources should be as one important measuring resource in combination with relatedness and growth resources.

Affect-based needs are categorized into formal and informal affect-based needs, referring to the emotional connection in the formal organization relation and informal or private relations respectively (e.g., working relationship, sponsorship, friendship, companionate love, etc.) [34,35]. Affect-based needs are informed by two emotional expressions in the complicated Chinese context, which are the assumed and real affections. The previous one is a psychological expression of obligations and regulation, and the latter is a spontaneous and inner psychological expression [36,37]. This study defines the affect-based needs experienced in an organizational context and characterized by the emotional expression guided by the obligations, regulation, and constraint of work roles in the formal organization as formal affect-based needs. In contrast, the other emotional expression of privacy, freedom, spontaneous, and reality in daily life beyond the boundaries of work roles is termed as informal affect-based needs.

**Figure 1:**
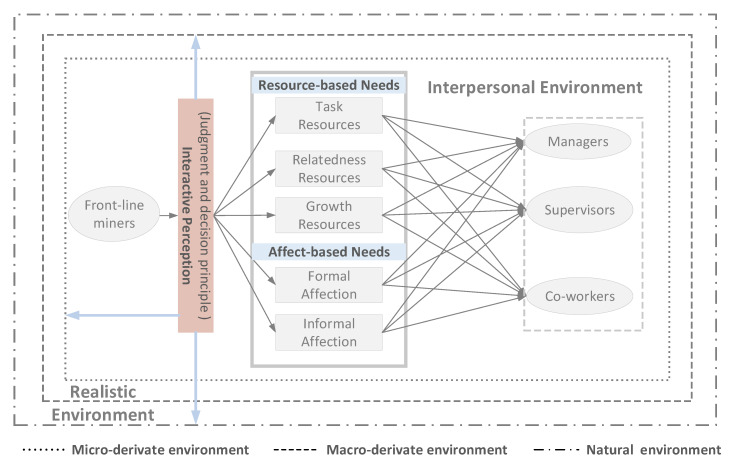
The structure of the psychological interaction of frontline miners in the coal mine production system.

In summary, the realistic (i.e., natural, macro–micro derivate environments) and interpersonal environment (the interaction with multi-roles like managers, supervisors, and co-workers based on the resource- and affect-based needs) constitute the basic components of the psychological interaction of frontline miners. The psychological interaction (1) of the above-mentioned elements further drive their behavior performance in the whole production system. The structure of psychological interactions of frontline miners was presented in Figure 1 based on the authors’ prior research and the above literature review.

This study explored the psychological interactions of frontline coal mine workers in China and the extent to which they satisfy their resource-based and affect-based needs. It is a first step in determining how these processes might increase behavioral risk, which will be investigated in later studies. The aims of the study were to:

(1) Identify and compare the interactive perceptions that frontline workers have toward their managers, supervisors, and co-workers with respect to resource-based (i.e., task, relatedness, growth) and affect-based needs (i.e., formal, and informal) and those associated with the realistic environment (i.e., natural, macro-derivate, micro-derivate).

(2) Identify how differences in demographics (age, marital status, no. of dependents, education, religion) and work characteristics (income, experience, accommodation) influence frontline miners’ perceptions of:

- Resource-based and affect-based needs provided by their managers, supervisors, and co-workers,
- The actual environment (i.e., natural, macro-derivate, micro-derivate).

(3) Identify the quadrant distributions of frontline miners (i.e., low affect/low resources; low affect/high resources; high affect/low resources; high affect/high resources); separately for managers, supervisors, and co-workers.

## 3. Research Design and Survey

### 3.1. Variable Measurement

A preliminary questionnaire was developed based on the literature key themes in the academic literature (job satisfaction [20], mental health [5], social environment and organizational environments [3]); the authors’ prior research [14,18,19]; the actual physical situations and real interactions between Chinese coal mining workers and their managers, supervisors, and co-workers; and expert interviews. Then, a pilot test was conducted that included 150 effective samples with a completion percentage of 92%. Modifications of the pre-survey questionnaire were made based on the reliability and validity analysis of Cronbach’s α (>0.7) and the item-to-total correlation (>0.3).

The final questionnaire consisted of 62 self-reported items. Eight items obtained basic individual information including the participants’ age, educational level, marital status, work experience, number of people/children supported, monthly income, family’s monthly income, and religion. The remaining questionnaire was the psychological interaction scale, which consisted of 54 items and measured the workers’ interactive perceptions of the actual environment, and their resource-based and affect-based needs. The workers’ resource- and affection-based interactions were measured separately for interactions with their manager/s (M-I), supervisor/s (S-I), and co-workers (W-I).

The actual environment interaction (E-I) measured the workers’ interactions with three dimensions: the natural environment (NE-I), the macro-derivative environment (MACRO-I), and the micro-derivative environment (MICRO-I). There were three items used to measure each dimension. Across dimensions, one item measured controllability, a second item measured adaptability, and a third item measured superiority. Examples of these are: “The natural issues were controlled fully in our mine” (i.e., controllability), “I completely adapted to the natural environment of our mine” (i.e., adaptability), and “The natural environment of our mine was extremely superior compared with others” (i.e., superiority).

Workers’ resource-based interactions (R-I) were measured with three dimensions: task resource interaction (TR-I), relatedness resource interaction (RR-I), and growth resource interaction (GR-I). Again, three items were used to measure each dimension, measured efficiency (e.g., “The task afforded by my Managers (Supervisors/Co-workers) was extremely timely”, utility (e.g., “The task aspect afforded by my Managers (Supervisors/Co-workers) was extremely appropriate and satisfied me”), and fairness (e.g., “The task aspect afforded by Managers (Supervisors/Co-workers) was highly fair and reasonable for me”.

Affect-based interactions (A-I) were measured with two dimensions: formal affection interactions (FA-I) and informal affection interactions (IA-I). Each had three items that measured the boundaries (e.g., “The relationship between me and my Managers (Supervisors/Co-workers) is the only job related”), support (e.g., “At work, my Managers (Supervisors/Co-workers) respect and support me very much”), and trustworthiness (e.g., “At work, I trusted my Managers (Supervisors/Co-workers) very much”) provided by the relationship.

Workers responded to all items using a ten-point Likert scale, and each item was scored from 1 to 10, where 1 indicated “highly disagree”, and 10 indicated “highly agree”. The same questionnaire was used for both groups of workers.

### 3.2. Research Process and Sample

Five researchers went to each of the six mine sites to distribute and collect the questionnaire. Workers completed the questionnaire individually prior to their shift. Researchers were present to assist those with low literacy skills. Managers were not present when workers completed the survey. Participants who completed the questionnaire online were recruited by distributing the web address electronically through a professional website. The procedures of this study were approved by the University of China University of Mining and Technology.

Participants were 642 Chinese coal mining workers, all of whom were male. Their demographic details are given (see Appendix A, Table A1). A total of 786 workers were given questionnaires, making an effective recovery rate of 84.5%. Nearly all of the participants (92%) were frontline workers from six underground mining companies operating in the northeastern (Mine A and Mine B), central (Mine C and Mine D), southeastern (Mine E), and western regions (Mine F) of China. Mining companies were run by Chinese provincial governments or the national government. Considering the reality of the numbers of coal mine companies distributed in the northeastern, central, and southeastern regions account for 93.23% of all Chinese companies [38], the sample was mostly in three regions. The number of participants and effective respondents in Mines A, B, C, D, E, F were 170/135, 140/109, 120/100, 110/92, 114/94, 70/50, respectively. The remaining participants were employed at coal mines across China who completed the questionnaire online, and the number of participants and effective respondents was 62/62.

### 3.3. Reliability and Validity Analysis

In this study, SPSS 22.0 and AMOS 21.0 were used for the statistical analysis of the questionnaire data. Notably, the negative items were used in the scale, so the paper converted those items to positive ones according to the consistency of the scale. First, the reliability of the questionnaire was tested with SPSS 22 based on Cronbach’s A and item-to-total (see Appendix A, Table A2).

The table shows that the reliability coefficient of the scale was adequate with Cronbach’s values for the factors ranging from 0.757 to 0.949, and the Cronbach’s alpha for the complete survey was 0.918. At the same time, the value of the item-to-total correlation was not less than 0.5 (>0.3), proving good validity as any item in the set of tests was consistent with the averaged behavior of the others.

A principal component analysis was conducted to determine the underlying factors in the survey items. The KMO value was 0.94, and Bartlett’s test of sphericity found it to be significant (*p* < 0.001). The results confirmed eight factors that together accounted for 85.98% of the variation (see Appendix A, Table A3). The cumulative per cent of rotation sums of squared loadings (CRSSL) of eight factors were 12.12 per cent, 23.54%, 34.96%, 45.87%, 56.20%, 66.51%, 76.45%, and 85.98%, respectively.

After the exploratory factor analysis, separate factor analysis was conducted on E-I and R-I/A-I items using Amos 21 to analyze all the indicators of construct validity. The results of the fitting indexes reached a better range after the mode of E-I was adjusted once, and the mode of R-I/A-I was adjusted twice. The indicators of E-I were CMIN: 91.604; CMIN/DF: 3.983, GFI: 0.978, SRMR: 0.03, RMSEA: 0.068, AGFI: 0.938, NFI: 0.965; TLI: 0.978; CFI: 0.978, IFI: 91.604 and the indicators of R-I/A-I were CMIN: 523.233; CMIN/DF: 6.976, GFI: 0.897, SRMR: 0.05, RMSEA: 0.097, AGFI: 0.839, NFI: 0.954; TLI: 0.945; CFI: 0.961, IFI: 0.961. All the above fit indicators showed the overall good fit of this model, providing support for the validity of the questionnaire.

In the above-described factor analysis, responses were collapsed across managers, supervisors, and co-worker items. We did undertake separate factor analysis across the three working groups and found the same five factor structure across groups, being TR-I, RR-I, GR-I, FA-I, and IA-I. This was consistent with the structure described above for non-environment items.

## 4. Results

### 4.1. Descriptive Statistics

#### 4.1.1. Overall Analysis

The mean (M) and standard deviation (SD) for all dimensions are shown in Table 1 as are the number and percentage of participants whose mean for the dimension/factor was below five, that is, under the midpoint of the 10-point Likert scale. Participants with scores lower than five on a particular factor had a worse view of that aspect of their work environment.

The overall mean ratings given by workers for their interactions with the realistic environment (M = 6.33), managers (M = 6.07), supervisors (M = 6.13), and co-workers (M = 6.61) showed that their interactions with their co-workers were perceived as the most positive and those with their managers as the least positive. Mean ratings for each of the factors for managers, supervisors, and co-workers were between 5.28 and 6.78, indicating they were in the average to the low-average range. The lowest mean ratings were for relatedness and growth interactions with managers (RR-I: M = 5.88, SD = 2.35; GR-I: M = 5.84, SD = 2.35) and relatedness interactions with supervisors (M = 5.28, SD = 2.49). Additionally, the percentage ratings under the midpoint of all environments and task resources with the manager as well as the relatedness resources with frontline miners were higher, showing a significant negative.

#### 4.1.2. Comparison Analysis

Paired sample *t*-tests were used to determine the differences in the participants’ perceptions of their managers, supervisors, and co-workers for all the resources and affection variables and the outcomes are shown in Table 2. The outcomes show that the participants’ mean ratings for their co-workers was significantly more positive than those given to their managers in all variables, TR-I: *t* (641) = −5.361, *p* = 0.000; RR-I: *t* (641) = −5.421, *p* = 0.000; GR-I: *t* (641) = −7.056, *p* = 0.000; FA-I: *t* (641) = −2.294, *p* = 0.000; IA-I: *t* (641) = −2.324, *p* = 0.000. Results also showed that the participants’ mean ratings for their co-workers were significantly more positive than those given to the supervisors in three variables: the relatedness resource variable (*t* (641) = −11.225, *p* = 0.000) and both affection variables (FA-I: *t* (641) = −3.321, *p* = 0.000; IA-I: *t* (641) = −4.850, *p* = 0.000). The comparisons between managers and supervisors showed that the participants’ mean ratings were more positive for supervisors on the task resource (TR-I: *t* (641) = −4.447, *p* = 0.000) and the growth resource (GR-I: *t* (641) = −6.112, *p* = 0.000) variables. However, the converse was true for the relatedness resource (RR-I: *t* (641) = 6.628, *p* = 0.000) and informal-affection (IA-I: *t* (641) = 2.721, *p* = 0.000) variables. There was no significant difference between the ratings given to managers and supervisors for the formal effect variable.

#### 4.1.3. Dimension Analysis

**Figure 2:**
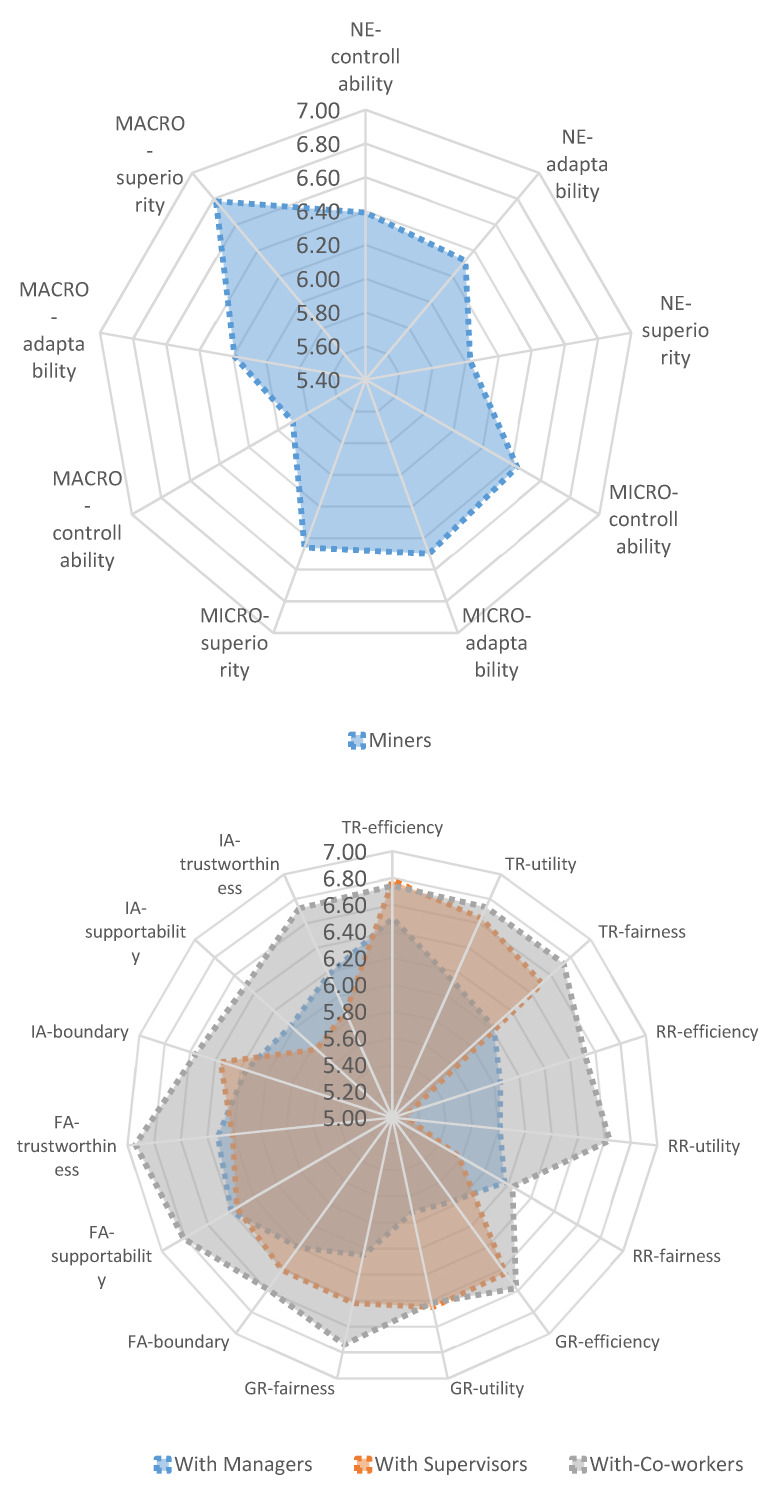
Spider graph of the total workers’ means for instrument items for the environment (macro, micro, natural environment), resource-based (task resources, relatedness resources, growth resources), and affect-based (formal affection, informal affection) interactions.

Spider graphs were used to plot the workers’ mean perception ratings for each of the individual items from the survey and are shown in Figure 2. The item ratings for the resource-based and affect-based dimensions were separated according to those given to managers, supervisors, and co-workers.

Means for the environmental interactions were between 5.90 and 6.78, and the lowest ratings were given to two items related to controllability in the MACRO-I dimension (M = 5.90) and superiority in the NE-I dimension (M = 6.03). As expected, the former item indicates that workers perceived they had only average control of the broader factors in their environment such as events influencing the stability of the coal mining industry. The latter result indicates that workers perceived the geological factors associated with the coal mine as being average. They were not in the high range, which indicated conditions meet the quality standards that support safe mining. The highest mean rating given to an environment item was for superiority in the MACRO-I dimension (M = 6.78), which indicated that workers perceived their industry and other broader factors were superior now compared to other periods.

Means for the resource-based and affect-based items for managers, supervisors, and co-workers ranged between 5.72 and 6.50, 5.13 and 6.78, and 6.04 and 6.94, respectively. Overall, the mean ratings given to co-workers were higher than those given to other roles. Compared with supervisors, the ratings given to TR-I and GR-I items were generally lower for managers. In contrast, ratings given to items from the RR-I dimension were higher for managers than supervisors, and ratings were similar for both worker groups for items in the FA-I dimension. There was some difference in ratings for items given within the IA-I dimension, with ratings being higher for managers than supervisors for trustworthiness and supportability items, but higher for supervisors than managers for the boundaries item. Thereinto, the lowest mean rating given to managers, was for utility in the GR-I dimension (M = 5.72). The lowest mean ratings given to supervisors were for supportability in the IA-I dimension (M = 5.77) and efficiency in the TR-I dimension (M = 5.78). Additionally, the lowest mean rating for co-worker was for fairness in the RR-I dimension (M = 6.04).

### 4.2. Difference Analysis of Single Variables

The means and standard deviations for each of the ratio variables for the realistic environment, managers, supervisors, and co-workers are shown in Table 3. One-way ANOVAs, followed by post hoc tests, were used in order to explore the different impacts of the social demographic variables. The omnibus ANOVA results are also included in Table 3 and show that there were significant differences within the levels of all variables.

With respect to the workers’ age, the mean score of frontline miners aged over 51 years old was significantly lower than others for E-I, M-I, W-I (E-I: 36–40 yrs (*p* = 0.037); 41–45 yrs (*p* = 0.004); 46–50 yrs (*p* = 0.003); M-I: <30 yrs (*p* = 0.037); 31–35 yrs (*p* < 0.001); 36–40 yrs (*p* < 0.001); 41–45 yrs (*p* < 0.001); 46–50 yrs (*p* = 0.001); W-I: <30 yrs (*p* = 0.001); 31–35 yrs (*p* = 0.011); 36–40 yrs (*p* = 0.005); and 41–45 yrs (*p* = 0.008)).

In terms of the monthly income, the frontline miners with an income of 6000–8000 RMB showed significantly higher mean scores for E-I, M-I, W-I than the others (E-I: ≤2000 RMB (*p* = 0.009); 2000–4000 (*p* = 0.000); 4000–6000 (*p* = 0.000); M-I: 2000–4000 (*p* = 0.000); ≤2000 (*p* = 0.008); 4000–6000 (*p* = 0.021); S-I: 2000–4000 (*p* = 0.000); W-I ≤2000 (*p* = 0.003); moreover, the ratings of workers with incomes between 2000–4000 for M-I were significantly lower than the ratings for those earning between 4000–6000 RMB (*p* = 0.001) and 6000–8000 RMB (*p* = 0.010).

In terms of monthly household incomes, the mean ratings of workers with household incomes between 5000–10000 RMBs were significantly higher for E-I, M-I and S-I than the mean scores of the others (E-I: with ≤3000 (*p* = 0.000); M-I: ≤3000 (*p* = 0.000); 3000–5000 (*p* = 0.000); S-I: ≤3000 (*p* = 0.000)). One other significant difference existed between the workers with household earnings between 10,000–20,000 RMB and <5000 RMB, with mean scores for S-I of the former group being significantly higher than that of the latter (*p* = 0.000).

In terms of the number of dependent people that workers were responsible, there was a significant difference in mean ratings given to the E-I dimension between workers with four and more than four dependents (*p* = 0.045), the latter was higher.

For household type, the mean scores for M-I for miners living in rental housing were significantly lower than it was for miners living in the surrounding countryside (*p* = 0.014). The mean score for S-I for miners living in rental housing was significantly lower than it was for miners living in group quarters and self-purchased commercial housing (S-I: group quarters (*p* = 0.040); self-purchased commercial (*p* = 0.007)).

Differences existed in the mean scores of miners with different levels of education as follows. Miners with only a junior school education had scores that were significantly lower on E-I, M-I, S-I, and W-I than that with other miners (E-I: junior college (*p* = 0.004); M-I: high school (*p* = 0.000); junior college (*p* = 0.005), senior technical (*p* = 0.035); S-I: junior college (*p* = 0.049); undergraduate (*p* = 0.015); postgraduate (*p* = 0.000); and W-I: junior college (*p* = 0.012); postgraduate (*p* = 0.012). Additionally, for E-I, there was a significant difference between the mean scores of miners with a junior college and postgraduate education (*p* = 0.001); the latter was lower than the mean score. For S-I, the miners with a primary school education had significantly lower mean scores than miners with postgraduate education (*p* = 0.024).

Finally, the analysis of mean score differences according to work experience showed that miners with 3–5 years of work experience had significantly lower mean scores for E-I than workers with 20–30 years of experience (*p* = 0.036). However, this was the opposite for S-I, as miners with 3–5 years of experience had higher mean scores than the other groups (5–10 yrs, *p* = 0.009; 10–15 yrs, *p* = 0.008; 15–20 yrs, *p* = 0.043; 20–30 yrs, *p* = 0.008).

### 4.3. Four-Quadrant Analysis

**Figure 3:**
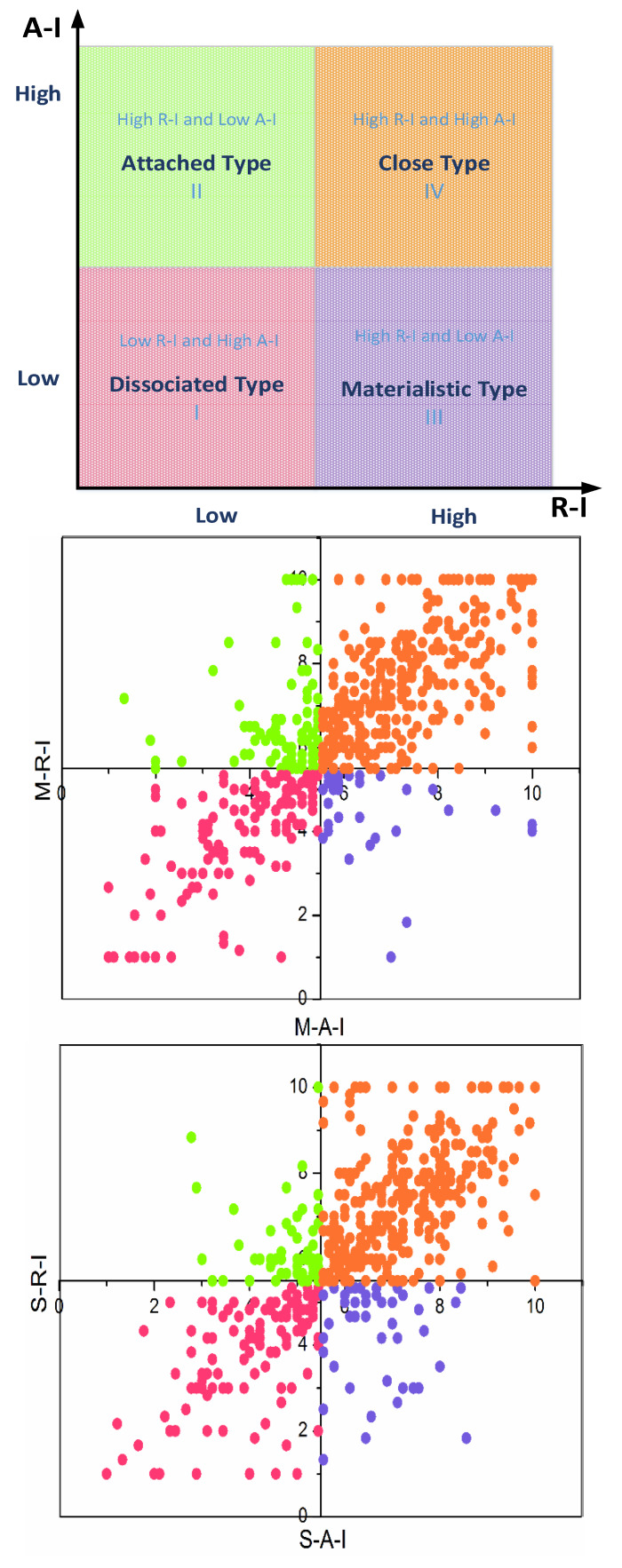

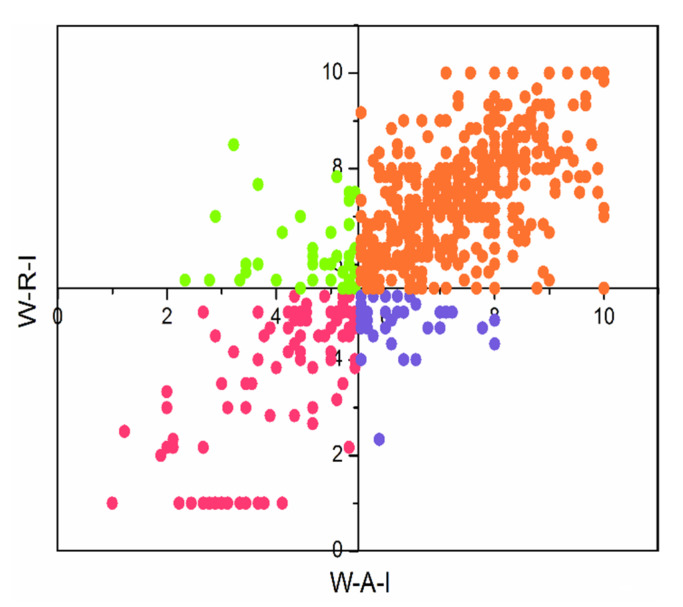
The four-quadrant analysis of the resources interaction (R-I) and affection interaction (A-I).

The four-quadrant was classified by the median of R-I and A-I, which represented four areas in Figure 3 (e.g., the low R-I and low A-I; low R-I and high A-I; high R-I and low A-I; high R-I and low A-I). The interpretations of all areas were shown as follows:

Dissociated type: Miners of this type had low R-I and A-I, in which the miners easily destroyed their relationship with other roles. This kind of miner easily takes a passive and negative attitude toward their work. The type of miners is not satisfied with work, and they will become a dangerous group for safety and health in enterprises.

Attached type: Miners connect the relationship with other roles by a strong affection, but their perception of R-I was greatly low. In the enterprises, some older employees often showed this trait, who were dedicated and loyal to enterprises, even with fewer resource returns. However, this type of miner cannot provide long-term safety guarantees.

Materialistic type: Miners connect the relationship with other roles by a strong resource exchange, but their perception of A-I is very low, which is the opposite to the attached type. This type of miner can easily cause unsafe outcomes or less safety commitment due to the lack of emotional strings.

Close type: Miners had a high perception of R-I and A-I with the others, which indicated the resources and affection involved could satisfy their needs to build a closer relationship and adopt altruistic behavior. This kind of miner is more stable for integral interests or safety development compared with other types.

The distribution of the four-quadrants I, II, III, and IV for M-I was 24.45%, 16.98%, 5.61%, 53.74%, respectively. The distribution of the four-quadrants I, II, III, and IV for S-I was 23.99%, 10.59%, 9.35%, 56.70%, separately. Likewise, that of W-I was 17.13%, 7.32%, 6.54%, 69.63%, respectively. To sum up, the order of the distribution was III < II < I < IV, and although the distribution of IV was the largest and the distribution of I was the second, showing a polarized status. In the above figures, the close type of miners for W-I was more than that for the other roles’ interaction, which indicated that the relationship between co-workers was stronger than that of the managers or supervisors.

## 5. Discussion

Miners are the basic implementers of coal mine production, occupying the main proportion of all personnel in coal mining companies. They are direct actors that trigger risky behavior so that their poor perception of psychological interaction will easily destroy the normal stability of safe and healthy working [8].

The results of the survey indicated that the miners had low environmental interaction, especially the controllability of all environmental dimensions. The reasons can be found that current external environments are really not optimistic. The working environment of the mine is worse such as high temperature, high humidity, dust, radiation, etc. [39]. Additionally, with the decrease in China’s high quality in the shallow layer of coal resources, the mining has gradually extended to the deep underground (even more than 1000 m), which causes more complex and serious hazards such as frequent roof fall and water inrush, etc. [15,40]. Furthermore, this nearly enclosing deep mine and high-intensity labor in unit time can easily cause physical damage and psychological repression of the miners [41]. Likewise, the macro-derivative environment is not optimistic since the policy of coal de-capacity is still the primary aim in the long term, where the coal miners are not only faced with the loss of unemployment, but also the decline of their identification and social position [42]. At the same time, the rights of safety management cannot be guaranteed to cause serious collusion between government regulators and firms, ignoring the real appeals of miners, resulting in their hopeless and helpless prospects [43]. Aside from this, the system of coal mining companies (e.g., production, management, and promotion system) is not perfect due to the concerned absence of poor physiological status and dealing with the coal transition [44,45].

The multi-role interaction also showed a low level. Thereinto, the dissociated type of M-I, S-I, and W-I was second highest, except for the closed type, which proved that a disharmonious relationship really exists within the coal mine production system. This part of the result is similar to the findings by Jiskani et al., who pointed out that the rank of miners perceived lower levels of supervisor safety, co-worker safety, and job safety [46]. A good relationship can create a strong power for development [47]. Specifically, as for S-I, the contradictory relations between supervisors and miners exist due to the opposite interests, that is, the supervisors’ investigation is directly linked to their performance, but it makes miners pay a high penalty [22]. Undeniably, the supervisors had an absolute right so the rent-seeking was widespread. The current supervision only comes from the superior leaders, and the real voices and problems from miners are easily ignored [48,49]. Furthermore, it was found in the field survey that supervisors and team leaders illegally selected scapegoats to be violators monthly to avoid offences and reduce workload. On the miner side, they usually cannot control their emotion by themselves, so that has caused serious reactions such as foul language, omission, brawls, and revenge, etc. For the M-I, the task resources and growth resources were relatively weaker than the other interactions. There was no direct conflict between the miners and managers like supervisors. However, the managers’ decisions generally protected the interests of the enterprise or themselves, and not the grassroot miners, which results in the miners’ tasks and growth resources not being committed. The history and culture of identification distances and hierarchy oppression has formed in the long run, which makes the miners have a sense of mistrust for managers [50,51]. Moreover, the history and culture of coal mine management is characterized by identification distances due to the different rights and hierarchy oppression and so on, which makes the miners have a sense of mistrust for managers and always keep silent resentment instead of expressing directly and going on their original risky way [50,51,52]. In the results, the special items of the fairness of task resources and the utility of all resources were especially low. The phenomenon also stated an implicit rule that resource allocation was based on a close or distant private relationship. Furthermore, some studies have proposed that the lack of transparency has resulted in widespread suspicion and concern of the miners regarding fairness [53]. The low utility illustrated that the resources (e.g., salary, tools, equipment, communication, materials and financial help in life, participated right, promotion, transfer position, and expenditure, etc.) did not meet the miners’ real needs, especially that regarding salary. The income of miners was reduced. Obviously, the interviewees’ salaries were mostly between 2000 and 4000 a month, which is a huge gap compared with that of more than 10,000 in the golden period of coal mining in China, and is also lower than the 6007 RMB monthly salary in manufacturing (72,088 RMB yearly) and similar to that of the 4021 RMB monthly salary in accommodation and catering (48,260 RMB yearly) as reported by the National Bureau of Statistics of China [1]. Meanwhile, the family burden of miners is very high, as they generally need to raise three to five people including children and the elderly, producing a huge psychological pressure on the miners [54]. A total of 70% of miners lived in rural areas, renting, or collective houses and only 30% of miners could pay for a commercial estate. To sum up, while the long-term needs of miners cannot be met and their rights cannot be guaranteed, the miners were prone to the appearance of psychological exhaustion, which accelerates the tendency of unsafe behaviors [55,56].

Many interesting outcomes by the difference analysis of single variables were obtained such as the extreme phenomenon and differentiated perception for multi-roles. The eldest had the lowest interactive perception for the E-I, M-I, S-I, and W-I, which is consistent with the research conclusions of Steverink et al., who put forward that the aging process was related to a changing balance between gains and losses, and they were less positive as they increased in age [57]. Furthermore, the miners with high monthly incomes or household earnings did not mean a higher perception of E-I, M-I, S-I, and W-I. The frontline miners with monthly incomes of 6000–8000 or household earnings of 5000–10,000 had relatively higher perception. These results did not accord with the study by Ng and Diener, who agreed that the higher income of people had a more positive psychological state and well-being [58]. The growing number of people raised as miners was positive with their perception. The reason may be that the appropriate pressure indirectly improves their tolerance of the external environment. Notably, miners with good living conditions benefited from psychological perception considering a greater sense of security and stability through houses in the Chinese cultural context, there, the group quarters and self-purchased commercial housing were better than the rental houses. Regarding education, the frontline miners with JSS showed a relatively low value than others for E-I and M-I, S-I, and W-I, which agreed with the study by Bjursell et al., who pointed out that education was positive with the work [59]. In contrast, frontline miners with the education of GR showed a low perception of E-I.

## 6. Conclusions

This paper explored the psychological interaction perception of miners based on the basic component elements in the coal mine production system, for example, the environment (natural environment, micro-derivate environment, and macro-derivate environment) and staff (managers, supervisors, and co-workers). This study reports a low interaction level of miners for the above elements, which can easily trigger unsafe behavior and even accidents in coal mines, and hinders the health and sustainable development of coal mine companies. The specific results of this study are as follows:

The interactive perception was classified into four objects: Environment interaction (E-I), Manager interaction (M-I), Supervisor interaction (S-I), and Co-worker interaction (W-I) while eight factors (i.e., the natural/macro-derivative/micro-derivative environment interaction, NE-I/MACRO-I/MICRO-I), task/relatedness/growth resource interaction (TR-I/RR-I/GR-I), and formal/informal affection interaction (FA-I/IA-I).

The E-I had the highest proportion of inferior values, followed by M-I. The rank of multi-role interaction was generally S-I < M-I < W-I, but the TR-I and GR-I with managers were the worst. Moreover, the IA-I was weaker than that of FA-I for all subjects. The miners generally had a lower perception of the controllability for all dimensions of E-I. Regarding the interaction between multi-roles, fairness, utility, and supportability were relatively lower, and the boundary was highest except for W-I.

Single demographic variables showed differences in multi-roles. Age, monthly income, monthly family income, education, the number of raised people, housing type, and work experience both showed significant differences for E-I and M-I, S-I, W-I except for the single demographic of marital status and religion.

The four types of miners were classified based on the four-quadrants: the dissociated type; attached type; materialistic type; and close type. Notably, the W-I showed a better relationship than that of M-I and S-I (highest distribution of close type). M-I and S-I revealed a high distribution of dissociated type after the close type, which showed a polarized status.

This study has the following implications based on the previous results from the perspective of the health psychology concept, environment, managers, supervisors, and miners in a coal mine safety production system. First, establish the cultural basis of the health psychology interaction system. The psychological interaction of miners should be given attention, and a psychological assessment information system should be built to report the psychological issues in a timely manner. Second, special members should be gain more attention and care. For example, the extreme individuals (e.g., the miners of youngest, eldest, highest, and lowest highest income, education, dissociated type) and different treatment methods should be adopted in accordance with their different needs. Third, the perception of environment interaction should be improved such as the upgrade in technology and equipment, safety monitoring system, and completing the working system of production, development, management, and culture. Fourth, clarify the responsibilities and perform different duties for managers and supervisors (e.g., the investment of the task, relation, and growth resources, the responsibility of role model, transparent and equal decision, timely communication and feedback, affection caring and so forth). Fifth, improve the ability and quality of miners such as a sense of duty and regulate pressure resistance, working communication, and team coordination.

Although the results of this study provide some important references for the safety and health management of coal mines, there are still some shortcomings that require further research. As a case study, part of the analysis focused on the intuitive description. In addition, the questionnaire design of the frontline miners’ psychological perception was limited since the coal mining system is complex, and previous mature psychological theories about miners are relatively rare. Moreover, a future study will adopt a big data approach to reveal the miners’ psychological perception map, which greatly improves the interpretability of related study. Additionally, time evolution of psychological perception based on the intervention experiments should be conducted to improve the effectiveness of policies in the future.

## Figures and Tables

**Table 1:** The mean value, standard deviation (SD), and frequency and percentage of ratings under the midpoint for each dimension.

Dimension			Factor			Ratings under the Midpoint	
Label	M	SD	Label	M	SD	N	%
E-I	6.33	1.83	NE-I	6.25	2.34	140	33.33
			MACRO-I	6.47	2.35	116	47.66
			MICRO-I	6.29	2.07	101	48.13
M-I	6.07	1.86	TR-I	6.21	2.32	135	51.40
			RR-I	5.88	2.35	148	34.42
			GR-I	5.84	2.47	171	38.01
			FA-I	6.31	2.23	108	22.74
			IA-I	6.12	2.31	125	23.99
S-I	6.13	1.81	TR-I	6.64	2.30	103	26.01
			RR-I	5.28	2.49	212	44.39
			GR-I	6.44	2.24	106	27.10
			FA-I	6.32	2.27	110	29.28
			IA-I	6.00	2.26	157	35.36
W-I	6.61	1.65	TR-I	6.73	2.01	80	22.27
			RR-I	6.40	2.03	93	26.48
			GR-I	6.58	2.11	81	26.17
			FA-I	6.78	1.93	63	19.94
			IA-I	6.58	1.97	82	29.60

Note: M-I represents the miners’ interaction with managers, S-I represents the miners’ interaction with supervisors, and W-I represents the miners’ interaction with co-workers. Inferior value frequency/percentage is the number/percentage of participants who responded with a rating lower than the mid-point of 5. The Likert scale used was 1 is “highly disagree” and 10 is “highly agree”; lower scores indicate a more negative perception of the dimension/factor.

**Table 2:** The paired sample statistics for multi-roles.

Subjects		M-S	M-W	S-W
TR-I	Mean Differences	−0.426	−0.517	−0.091
	*t*	−4.447 ***	−5.361 ***	−0.987
RR-I	Mean Differences	0.603	−0.524	−1.127
	*t*	6.628 ***	−5.421 ***	−11.225 ***
GR-I	Mean Differences	−0.596	−0.736	−0.14
	*t*	−6.112 ***	−7.056 ***	−1.489
FA-I	Mean Differences	0.114	−0.199	−0.313
	*t*	1.299	−2.294 ***	−3.321 ***
IA-I	Mean Differences	0.12	−0.462	−0.582
	*t*	2.721 ***	−2.324 ***	−4.850 ***

Note: M-S represents the paired differences on the perception of frontline miners for managers and supervisor and, by extension; *** *p* < 0.001.

**Table 3:** Means (SDs) for socio-demographic variables for the realistic environment, managers, supervisors, and co-workers’ interactions.

Variables	Classifications	E-I	M-I	S-I	W-I
Age	<30	6.04 ± 1.55	6.01 ± 1.80	/	6.94 ± 1.55
	31–35	6.36 ± 1.63	6.30 ± 1.78	/	6.76 ± 1.59
	36–40	6.44 ± 1.83	6.47 ± 1.92	/	6.29 ± 1.59
	41–45	6.57 ± 1.72	6.27 ± 1.78	/	6.71 ± 1.51
	46–50	6.63 ± 1.98	6.26 ± 1.97	/	6.59 ± 1.55
	>51	5.44 ± 2.26	4.87 ± 2.39	/	5.69 ± 2.32
Sig.	/	4.88 ***	6.74 ***	/	5.70 ***
Monthly income (RMB)	≤2000	6.12 ± 1.74	5.98 ± 1.85	5.91 ± 1.60	6.15 ± 1.57
	2000–4000	6.08 ± 2.01	5.62 ± 2.11	5.78 ± 1.84	6.24 ± 1.86
	4000–6000	6.19 ± 1.53	6.31 ± 1.70	6.24 ± 1.75	6.74 ± 1.54
	6000–8000	7.27 ± 1.56	6.90 ± 1.65	6.85 ± 1.58	7.18 ± 1.17
	8000–10,000	7.12 ± 2.31	7.36 ± 1.99	6.82 ± 2.10	7.24 ± 1.84
	>10,000	5.35 ± 1.34	5.96 ± 2.07	5.49 ± 1.62	5.61 ± 1.37
Sig.		8.39 ***	9.22 ***	6.67 ***	7.11 ***
Monthly household earnings (RMB)	≤3000	5.79 ± 1.95	5.64 ± 2.26	5.78 ± 1.74	6.07 ± 1.92
	3000–5000	6.27 ± 1.88	5.87 ± 1.93	5.94 ± 1.82	6.53 ± 1.57
	5000–10,000	6.73 ± 1.62	6.67 ± 1.66	6.38 ± 1.74	6.83 ± 1.54
	10,000–20,000	6.37 ± 1.87	6.21 ± 1.73	6.87 ± 1.77	7.09 ± 1.43
	>20,000	6.22 ± 1.75	6.21 ± 2.42	6.21 ± 1.72	5.78 ± 2.27
Sig.		5.39 ***	7.26 ***	5.11 ***	6.45 ***
Number of dependent people	1	6.17 ± 1.98	/	/	/
	2	6.08 ± 1.43	/	/	/
	3	6.72 ± 1.83	/	/	/
	4	6.22 ± 1.99	/	/	/
	>4	6.30 ± 1.71	/	/	/
Sig.	/	2.91 *	/	/	/
Housing type	Miner village	/	6.33 ± 1.84	6.12 ± 2.05	/
	Rental housing	/	5.92 ± 2.22	5.71 ± 1.57	/
	Group quarters	/	6.04 ± 1.60	6.44 ± 1.63	/
	Surrounding countryside	/	6.62 ± 1.76	5.66 ± 1.93	/
	Self-purchased commercial housing	/	5.89 ± 1.95	6.43 ± 1.68	/
	Others	/	6.07 ± 2.03	6.10 ± 1.04	/
Sig.		/	2.69 *	5.14 ***	/
Education	<PS	7.33 ± 2.28	6.91 ± 1.94	6.72 ± 1.96	6.83 ± 1.47
	PS	6.71 ± 1.70	6.52 ± 1.70	5.84 ± 1.82	6.24 ± 1.60
	JS	6.03 ± 1.99	5.69 ± 2.17	5.74 ± 1.86	6.29 ± 1.86
	HS; ST	6.51 ± 1.74	6.43 ± 1.76	6.26 ± 1.70	6.66 ± 1.58
	JC	6.93 ± 1.65	6.61 ± 1.79	6.39 ± 1.60	6.92 ± 1.52
	U	5.97 ± 1.47	6.14 ± 1.59	6.62 ± 1.70	6.74 ± 1.35
	P	5.74 ± 1.34	5.75 ± 1.26	8.14 ± 0.81	7.47 ± 0.92
Sig.		4.78 ***	4.57 ***	6.82 ***	3.16 ***
Work experience	<3 years	7.06 ± 1.80	/	6.58 ± 1.64	/
	3–5 years	5.85 ± 1.24	/	7.11 ± 1.54	/
	5–10 years	6.11 ± 1.65	/	5.98 ± 1.58	/
	10–15 years	6.39 ± 1.92	/	5.93 ± 1.79	/
	15–20 years	6.60 ± 2.01	/	6.14 ± 2.16	/
	20–30 years	6.63 ± 1.84	/	6.04 ± 1.75	/
	>30 years	5.81 ± 2.33	/	6.17 ± 1.85	/
Sig.		3.04 ***	/	3.73 ***	/

Note: E-I = Realistic environment interactions, M-I = Manager interactions, S-I = Supervisor interactions, W-I = Co-worker interactions; Education: PS—Primary school; JS—Junior school; HS—High school; ST—Secondary technical; JC—Junior college; U—undergraduate; P—postgraduate; * *p* < 0.05, *** *p* < 0.001.

## Appendix A

**Table A1:** The frequency and percentage of social demographic variables of all participants.

**Age**	<30	98	15.26	**Monthly family income (yuan)**	≤3000	125	19.47
	31–35	108	16.82		3000–5000	237	36.92
	36–40	100	15.58		5000–10,000	211	32.87
	41–45	153	23.83		10,000–20,000	51	7.94
	46–50	119	18.54		20,000–50,000	15	2.34
	>51	64	9.97		>50,000	3	0.47
**Monthly household earnings (yuan)**	≤2000	57	8.88	**Work experience**	<3 years	23	3.58
	2000–4000	259	40.34		3–5 years	57	8.88
	4000–6000	197	30.69		5–10 years	176	27.41
	6000–8000	105	16.36		10–15 years	147	22.90
	8000–10,000	18	2.80		15–20 years	103	16.04
	10,000–20,000	4	0.62		20–30 years	105	16.36
	>20,000	2	0.31		>30 years	31	4.83
**Housing type**	Miner village	58	9.03	**Religion**	None	558	86.92
	Rental housing	52	8.10		Buddhism	32	4.98
	Group quarters	148	23.05		Christianity	14	2.18
	Surrounding countryside	174	27.10		Islam	21	3.27
	Self-purchased commercial housing	202	31.46		Taoism	12	1.87
	Others	8	1.25		Others	5	0.78
**Education**	<PS	7	1.09	**The number of supported people**	1 people	30	4.67
	PS	28	4.36		2 people	58	9.03
	JS	261	40.65		3 people	195	30.37
	HS; ST	151	23.52		4 people	193	30.06
	JC	113	17.60		>5 people	166	25.86
	U	69	10.75	**Work position**	Ordinary employees	446	69.47
	P	13	2.02		(deputy) group leader	103	16.04
**Marital status**	Unmarried	33	5.14		Group Supervisor	24	3.74
	Married	566	88.16		(vice) Captain	31	4.83
	Divorced	21	3.27		Others	38	5.92
	Re-married	22	3.43				

Note. Education: PS—Primary school; JS—Junior school; HS—High school; ST—Secondary technical; JC—Junior college; U—undergraduate; P—postgraduate.

**Table A2:** Reliability and validity analysis result of each variable of initial scale.

Scales	Classification	Factor	Cronbach’s Alpha	Item-to-Total
**Interaction measures**	Environment interaction (E-I)	Factor 1: NE-I	0.829	0.629~0.777
		Factor 2: MACRO-I	0.897	0.792~0.820
		Factor3: MICRO-I	0.757	0.512~0.736
	Resources interaction (R-I)	Factor 4: TR-I	0.936	0.840~0.901
		Factor 5: RR-I	0.896	0.702~0.850
		Factor 6: GR-I	0.949	0.870~0.901
	Affection interaction (A-I)	Factor 7: FA-I	0.916	0.753~0.872
		Factor 8: IA-I	0.901	0.718~0.853

**Table A3:** The factor loading matrix of psychology interaction.

Scale	Items	Component							
		1	2	3	4	5	6	7	8
**Factor 1 NE-I**	The natural issues were controlled fully in our mine.	**0.749**	0.072	0.243	0.294	0.287	0.108	0.228	0.194
	I completely adapted to the natural environment of our mine.	**0.758**	0.175	0.251	0.255	0.245	0.134	0.238	0.183
	The natural environment of our mine was extremely superior compared with others.	**0.689**	0.303	0.276	0.183	0.276	0.164	0.236	0.176
**Factor 2 MACRO-I**	The external environment issues were controlled completely.	0.264	**0.756**	0.329	0.096	0.297	0.029	0.116	0.097
	I extremely adapted to the external environment of coal enterprises.	0.258	**0.749**	0.336	0.157	0.272	0.002	0.099	0.127
	The current external environment of coal enterprises was highly unstable compared other periods. (R)	0.020	**0.882**	0.013	0.304	0.035	0.014	0.026	0.054
**Factor 3 MICRO-I**	The problems on the internal systems of my coal mine company was controlled fully.	0.259	0.383	**0.632**	0.273	0.296	0.169	0.208	0.175
	I completely adapted to all process and regulation of whole internal systems in our mine.	0.224	0.419	**0.669**	0.237	0.296	0.149	0.176	0.186
	The whole systems of my coal mine company were advanced in the extreme compared with others.	0.347	0.144	**0.672**	0.309	0.313	0.181	0.236	0.160
**Factor 4 TR-I**	The task afforded by managers/supervisors/co-workers was extremely timely.	0.352	0.061	0.434	**0.558**	0.356	0.213	0.210	0.149
	The task aspect afforded by managers/supervisors/co-workers was extremely appropriate and satisfied me.	0.377	0.016	0.460	**0.506**	0.365	0.208	0.224	0.174
	The task aspect afforded by managers/supervisors/co-workers was highly fair and reasonable for me.	0.169	0.302	0.349	**0.723**	0.230	0.092	0.146	0.137
**Factor 5 RR-I**	The daily relation aspect afforded by managers/supervisors/co-workers was extremely well-timed.	0.232	0.321	0.109	0.463	**0.653**	0.112	0.148	0.092
	The daily relation aspect afforded by managers/supervisors/co-workers was extremely appropriate and helpful to improve my relations.	0.258	0.246	0.288	0.306	**0.722**	0.103	0.102	0.165
	The better relation with managers/supervisors/co-workers, the more benefits got in daily life, and extreme injustice. (R)	0.320	0.152	0.310	0.330	**0.695**	0.153	0.154	0.143
**Factor 6 GR-I**	The career development aspect afforded by managers/supervisors/co-workers was extremely timely.	0.119	−0.020	0.121	0.186	−0.167	**0.799**	0.168	0.168
	The career development aspect afforded by managers/supervisors/co-workers was extremely appropriate and helpful for my growth.	0.078	0.009	0.071	0.081	0.177	**0.876**	0.165	0.098
	The career development aspect afforded by managers/supervisors/co-workers was highly fair and reasonable.	0.062	0.068	0.105	−0.017	0.303	**0.742**	0.322	0.073
**Factor 7 FA-I**	The relationship between I and my managers/supervisors/co-workers is only job related. (R)	0.203	0.030	0.124	0.195	0.046	0.291	**0.800**	0.148
	At work, my managers/supervisors/co-workers respect and support me very much.	0.191	0.102	0.187	0.135	0.103	0.272	**0.770**	0.250
	At work, I trusted my managers/supervisors/co-workers very much.	0.139	0.092	0.122	0.082	0.197	0.202	**0.793**	0.272
**Factor 8 IA-I**	The managers/supervisors/co-workers never told you about their private things. (R)	−0.059	0.462	0.052	−0.145	0.211	0.069	0.197	**0.709**
	The managers/supervisors/co-workers do their best to help me out of all difficulties whether in work or life.	0.100	−0.008	0.203	0.077	0.161	0.134	0.253	**0.833**
	The managers/supervisors/co-workers like brothers or relatives, and I extremely trusted them whether in work or life.	0.321	−0.089	−0.035	0.406	−0.123	0.128	0.078	**0.735**

Note. Bold values indicate the factor loading value is >0.5, reflecting the items of each factors. Items were translated from Mandarin to English and lost some meaning in the process; Factors 4 to 8 ask the same question with respect to three groups: managers, supervisors, and co-workers; (R) = reversed question.
