# Erratum: Han, S., et al. Who Reports Low Interactive Psychology Status? An Investigation Based on Chinese Coal Miners. *Int. J. Environ. Res. Public Health* 2020, *17*, 3446

**DOI:** 10.3390/ijerph18062853

**Published:** 2021-03-11

**Authors:** Shuai Han, Hong Chen, Ruyin Long

**Affiliations:** 1 College of Economic and Management, Shandong University of Science and Technology, Qingdao 266597, China; 2 School of Management, China University of Mining and Technology, Xuzhou 221116, China; longruyin@163.com

Ms. Jill Harris has requested that she should be removed from the list of contributing authors. After re-considering her contribution, we wish to remove her from the authorship of our paper [1] and add her contributions in acknowledgement. The updated “Author Contributions” and “Acknowledgments” are provided below: 
